# Moderate-Dose Local Radiotherapy in Symptomatic CD30-Positive, Anaplastic Lymphoma Kinase (ALK)-Negative Primary Cutaneous T-cell Lymphoma

**DOI:** 10.7759/cureus.104946

**Published:** 2026-03-09

**Authors:** Alejandra Varela, Sebastián Sepúlveda, Nicole E Evans, Rafael E Araúz, Liz M Champsaur, Elizabeth D Orcy, Osvaldo Samudio

**Affiliations:** 1 Faculty of Medicine, Universidad de Panamá, Panama, PAN; 2 Radiation Oncology, Instituto Oncológico Nacional, Panama, PAN; 3 Dermatology, Complejo Hospitalario Metropolitano Dr. Arnulfo Arias Madrid, Panama, PAN; 4 Hematology, Ciudad de la Salud, Panama, PAN

**Keywords:** alk-negative, case report, cd30-positive t-cell lymphoma, primary cutaneous t-cell lymphoma, radiation oncology, radiotherapy

## Abstract

Primary cutaneous anaplastic CD30-positive (+) T-cell lymphoma is a rare variant of primary cutaneous lymphoma characterized by proliferation of large T lymphocytes with strong CD30 expression and no anaplastic lymphoma kinase (ALK) expression. It typically presents as rapidly growing nodules or tumors with a tendency to ulcerate, but often has a favorable outcome in localized stages. Radiotherapy is an effective local treatment for cutaneous T-cell lymphomas, and several retrospective studies suggest that low-dose regimens may achieve high response rates with reduced toxicity, although prospective randomized data are lacking. We describe the case of a 65-year-old female patient who initially presented with generalized erythematous patches and plaques that progressed to painful nodules and later ulcerated. Biopsy confirmed a CD30+, ALK-negative, T-cell lymphoma. The patient received chemotherapy with the CHOEP regimen and, during the sixth cycle, developed a new ulcerated lesion on her right thigh accompanied by local signs of infection, leukocytosis, and fever. The patient was then started on antibiotics with no improvement. Localized radiotherapy was then administered, which resulted in progressive improvement of the lesion from the first sessions, with tumor volume reduction, decreased exudate, and partial re-epithelialization of the ulcerated bed. This case highlights the role of localized radiotherapy as an effective palliative and disease-controlling modality for cutaneous CD30+ T-cell lymphoma, particularly in symptomatic or treatment-refractory lesions.

## Introduction

Primary cutaneous anaplastic large cell lymphoma (PC-ALCL) is a subtype of non-Hodgkin lymphoma (NHL) within the spectrum of CD30⁺ T-cell lymphoproliferative disorders. It is histopathologically characterized by infiltrates of large T cells with prominent nuclear pleomorphism and CD30 expression in more than 75% of tumor cells [1]. The ALK-negative subtype is particularly rare, accounting for 6% of NHL cases, and is associated with a poorer prognosis than its anaplastic lymphoma kinase (ALK)-positive counterpart [1,2]. Cutaneous lymphomas predominantly affect middle-aged to older adults, typically in the sixth decade of life. Clinically, most patients present with solitary or multiple localized nodules with a tendency to ulcerate, most commonly involving the head, neck, and extremities [3]. Plaques and patches may also be present [4]. Due to the broad differential diagnosis, including other CD30+ cutaneous entities (mycosis fungoides, cutaneous B-cell lymphomas, angiosarcoma, viral infections, etc.), accurate diagnosis requires careful clinicopathologic correlation [1]. 

Management of PC-ALCL depends on the extent of the disease. Localized lesions are typically treated with surgical excision or radiotherapy, while multifocal disease may require systemic therapy. Radiotherapy plays an important role as an effective treatment modality for cutaneous lymphomas, as it can achieve rapid symptom relief and lesion regression with minimal toxicity. However, evidence supporting its use in PC-ALCL, particularly the ALK-negative subtype, remains limited due to the scarcity of randomized trials [3]. 

Reporting this case aims to provide practical evidence supporting localized moderate-dose radiotherapy in complex clinical scenarios where infection and ongoing systemic therapy limit other interventions, highlight diagnostic and multidisciplinary management challenges, and contribute real-world data to the literature by documenting clinical course, treatment response, and outcomes that can be pooled with other reports to inform future case series and prospective studies on dose selection and sequencing in PC-ALCL.

## Case presentation

**Figure 1:**
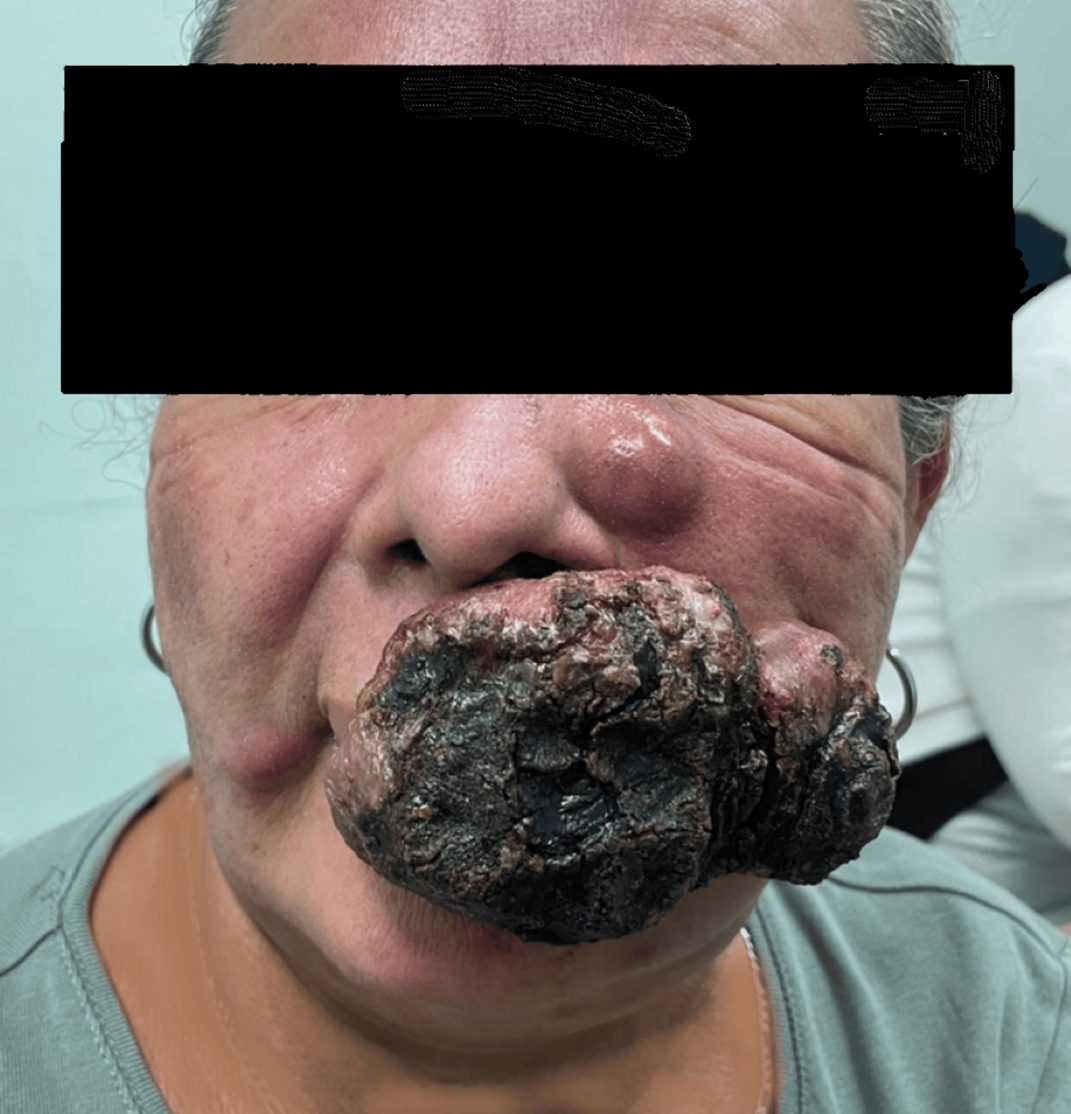
First lesion. Initial exophytic, necrotic tumoral mass in the perioral region extending to the left cheek.

A 65-year-old woman presented to the dermatology outpatient department of our hospital, Complejo Hospitalario Metropolitano Dr. Arnulfo Arias Madrid, with a six-month history of erythematous patches and plaques localized to the facial region. Over the preceding month, these lesions had evolved into painful erythematous nodules involving the cheeks and supralabial area. On physical examination, a large exophytic tumoral mass measuring approximately 6-7 cm was observed in the perioral region, extending to the left cheek. The lesion exhibited a broad base, poorly defined and infiltrated borders, and an irregular, ulcerated, friable surface. Multiple areas were covered with black necrotic crusts interspersed with erythematous to violaceous zones (Figure 1). Initially, the differential diagnosis included angiosarcoma and mycosis fungoides.

**Figure 2:**
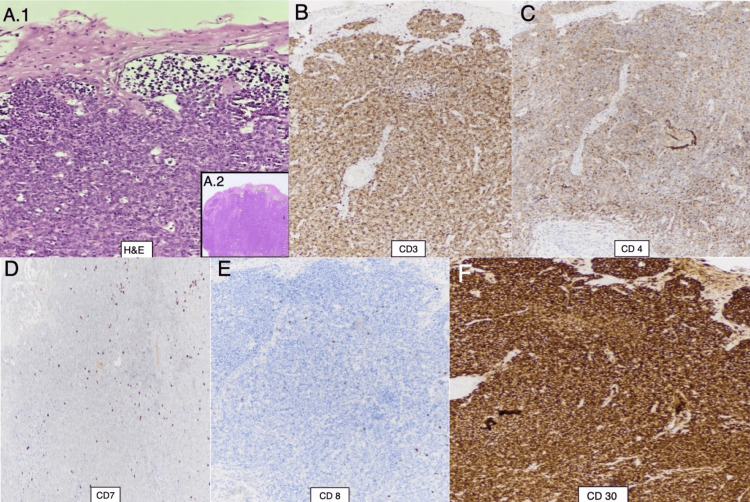
Histopathological and immunohistochemical findings. Hematoxylin and eosin-stained sections examined at high power (40x) reveal a diffuse proliferation of large pleomorphic discohesive cells with vesicular nuclei, prominent nucleoli, and frequent mitoses (A.1). At low power (4x), sections show ulceration and a dense subepidermal infiltrate (inset) (A.2). Immunohistochemical studies demonstrate that the neoplastic cell population is positive for CD3 (B) and CD4 (C), with strong, diffuse membranous and paranuclear expression of CD30 (D). The tumor cells show loss of CD7 expression (E) and are negative for CD8 (F).

Laboratory tests showed mildly elevated hemoglobin levels (15.6 g/dL; normal range: 12-15 g/dL) and normal levels of C-reactive protein (CRP) and erythrocyte sedimentation rate (ESR). Antinuclear antibodies (ANA), anti-double-stranded DNA antibodies (anti-dsDNA), and the Venereal Disease Research Laboratory (VDRL) test were all negative. An excisional skin biopsy of the right supralabial area showed ulcerated skin with epidermal atrophy and a dense subepidermal infiltrate of large pleomorphic cells characterized by eosinophilic cytoplasm, atypical nuclei, and frequent mitoses, within a background of necrotic debris and stasis. Immunohistochemistry revealed abundant cytoplasm positive for CD3, CD4, and CD30, and negative for CD20 and CD79a (Figures 2A-2F). Based on these findings, a diagnosis of primary cutaneous anaplastic large cell lymphoma (PC-ALCL) was established. Computed tomography (CT) showed no evidence of lymphadenopathy or solid lesions. The patient then started the first cycle of chemotherapy two weeks after her initial evaluation, with good tolerance and no complications. 

**Figure 3:**
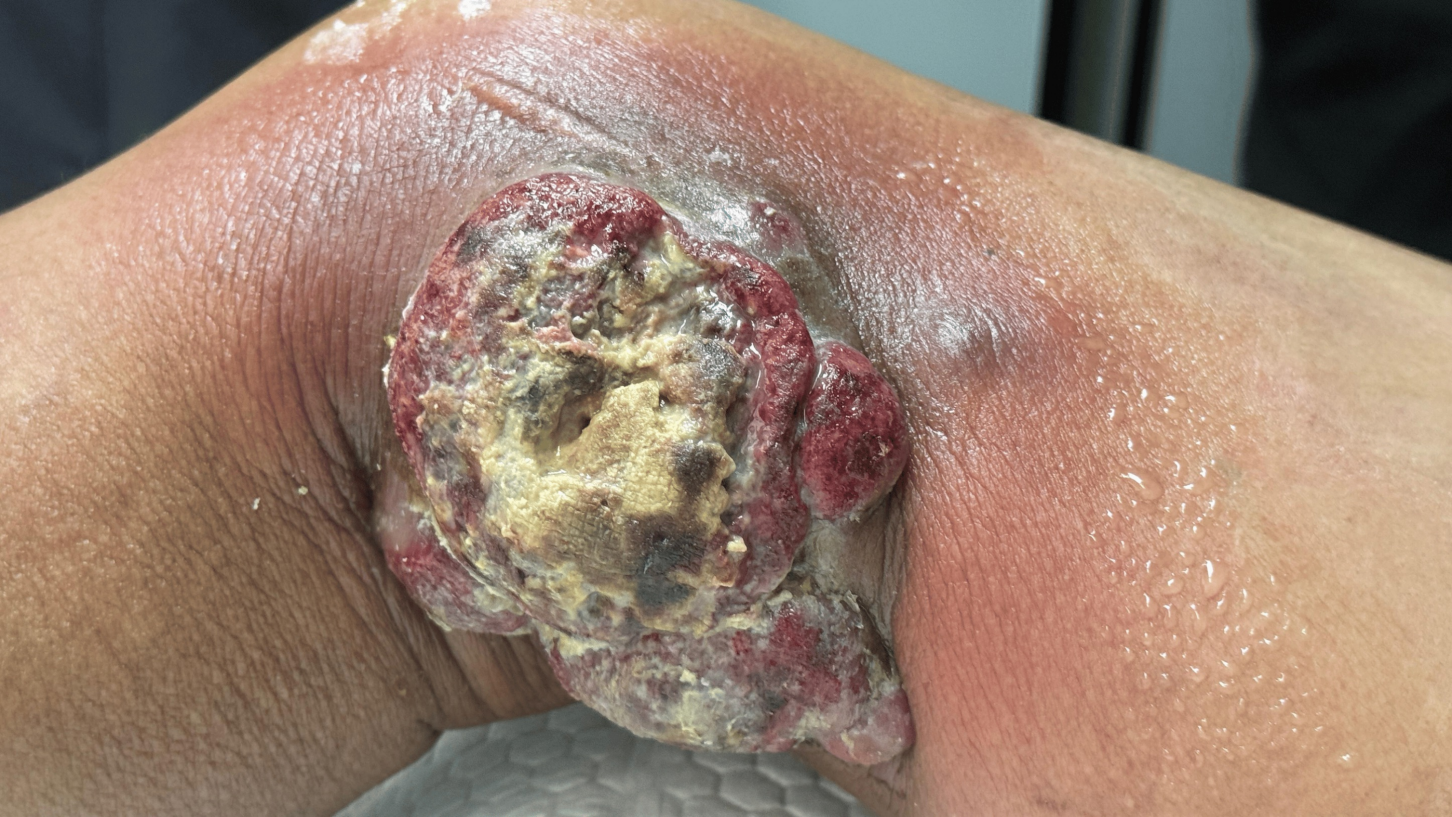
New lesion on the right thigh. Erythematous, exophytic, and fungoid-like lesion that appeared six months after the initial diagnosis.

Six months later, the patient presented to the outpatient clinic with a new painful ulcerous lesion on the medial side of her right thigh; she was referred to the radiation oncology service for further evaluation. Clinical examination revealed an erythematous, exophytic, serous fungoid-like lesion, measuring approximately 8 x 7 cm, with a malodorous discharge (Figure 3). A skin biopsy of the lesion demonstrated findings consistent with the initial biopsy, and findings consistent with multifocal cutaneous involvement of PC-ALCL were identified. Empiric antibiotic therapy was initiated with vancomycin and meropenem due to suspected bacterial infection. Radiation therapy with a regimen of 20 sessions with 36 Gy was started.

**Figure 4:**
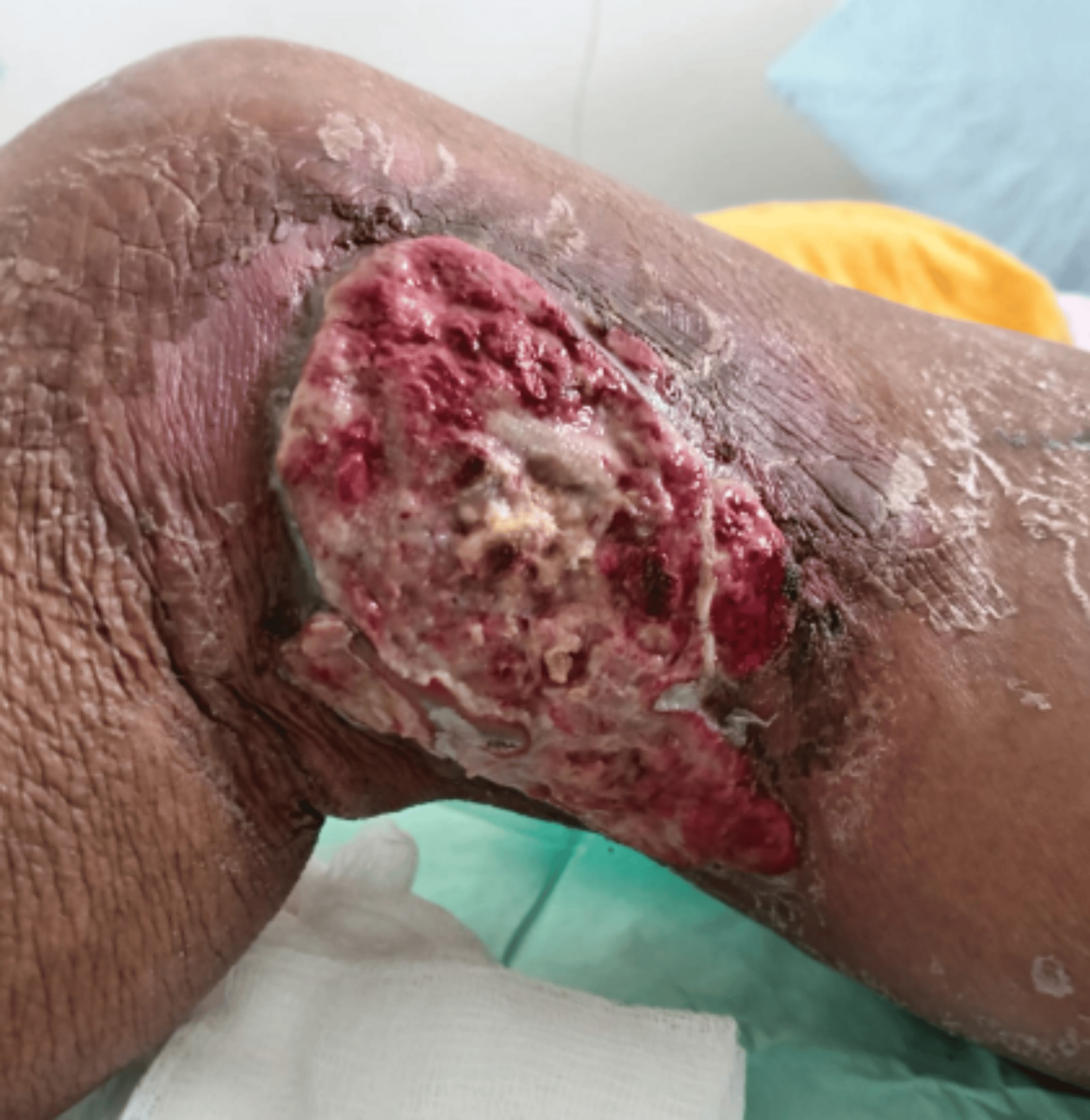
After the fourth radiotherapy session. Clinical improvement of the lesion with reduction in size and resolution of malodorous discharge.

**Figure 5:**
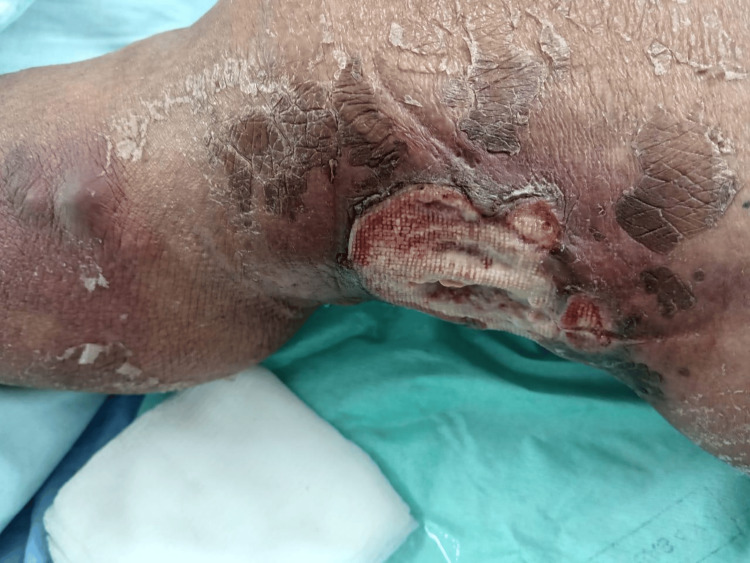
Twelfth radiotherapy session. Favorable progression of the lesion with the development of granulation tissue and increasingly well-defined borders.

**Figure 6:**
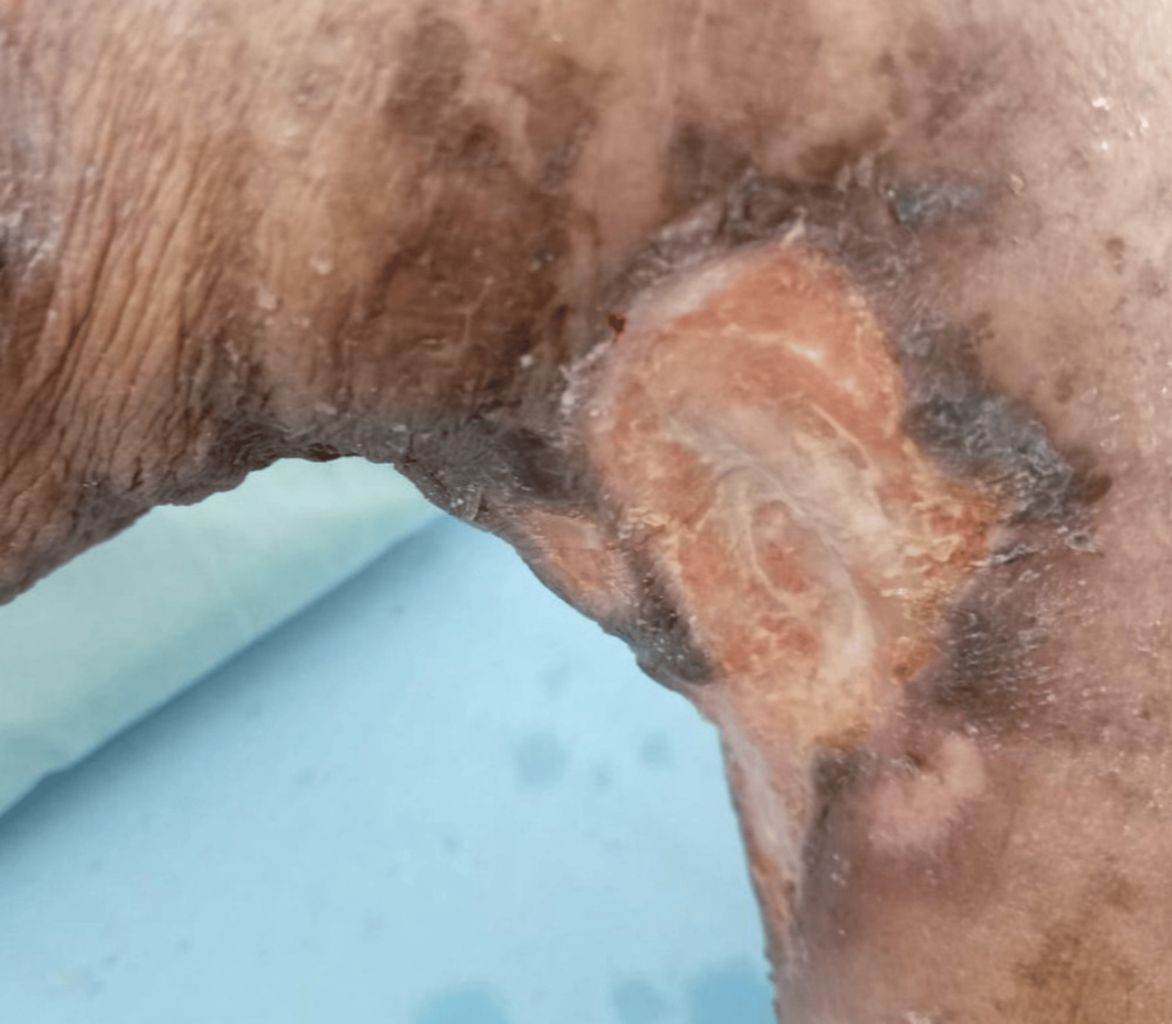
Last radiotherapy session. Favorable clinical evolution of the lesion with reduction in size, increased granulation tissue formation, and cicatrization.

Clinical improvement of the lesion was observed during the early fractions of radiotherapy. The patient had received approximately one week of antibiotic therapy before the initiation of radiotherapy, which may also have contributed to the improvement of local infectious signs (Figure 3). Initially exophytic, ulcerated, and malodorous, the lesion exhibited marked reduction in size, decreased exudate, resolution of the foul odor, and partial re-epithelialization of the ulcerated surface. This positive response progressed throughout the subsequent sessions, allowing early discontinuation of the antibiotic therapy due to a resolution of local signs of infection (Figures 4-6).

## Discussion

Approximately 25% of cutaneous T-cell lymphomas belong to the group of primary cutaneous CD30+ lymphoproliferative disorders, within which our case is classified as PC-ALCL. With a five year disease-specific survival (DSS) rate of 95%, patients suffering from this disease have an excellent prognosis [5]. Patients with PC-ALCL present ulcerating tumors or nodules, which are solitary or localized, and should be treated with Radiotherapy (RT) or surgical excision [5-6]. The diagnosis was made according to the World Health Organization (WHO) criteria, based on the presence of dense, nodular dermal infiltrates composed of large pleomorphic, anaplastic, or immunoblastic cells with large, irregularly shaped nuclei, abundant pale or eosinophilic cytoplasm, and expression of CD30 [7]. ALK expression is usually present in the systemic form of this disease but is uncommon in PC-ALCL [5].

Systemic chemotherapy with the CHOEP regimen has been evaluated in this setting. A pooled cohort analysis demonstrated a complete clinical response rate of 85%, although relapse rates were as high as 71% [8]. In patients with multifocal lesions treated with CHOEP-based combination therapy, complete response was achieved in 77% of cases and partial response in 33%; however, all patients eventually developed cutaneous relapses [9].

Radiotherapy has shown excellent outcomes in PC-ALCL. In a multi-institutional experience including 56 patients with stages ranging from T1a to T3b, radiation doses between 6 and 45 Gy (median dose 35 Gy) were administered, with a median fraction size of 2 Gy. Tumors treated with doses between 30 and 39 Gy achieved a complete clinical response rate of 97% [7]. In another cohort, radiotherapy achieved a complete response rate of 100% in 21 patients [9].

In our patient, the presence of two noncontiguous lesions corresponded to stage T3a according to the International Society for Cutaneous Lymphomas (ISCL) and European Organisation for Research and Treatment of Cancer (EORTC) TNM classification for primary cutaneous lymphomas [8]. Current EORTC recommendations suggest that, for multifocal lesions, a low dose of 8 Gy (2 × 4 Gy) may be sufficient, based on studies demonstrating no significant difference between low-dose (<20 Gy) and intermediate-dose (21-39 Gy) radiotherapy in achieving complete response [3,7,10].

However, lesion size appears to influence prognosis. Lesions larger than 5 cm have been associated with poorer local control and long-term outcomes, and relapsed lesions tend to be larger [8]. Although current EORTC guidelines do not explicitly incorporate lesion size into treatment selection for PC-ALCL, this represents an area requiring further investigation, particularly for higher-risk patients [3,11].

In our case, the patient presented with an 8 cm lesion and rapid disease progression. Given the limited evidence regarding optimal dosing in larger, higher-risk lesions, the use of moderate-dose radiotherapy (36 Gy in 20 fractions) was considered justified to maximize local control and reduce the risk of relapse.

## Conclusions

This case illustrates that localized radiotherapy may be an effective therapeutic option for symptomatic cutaneous CD30-positive lymphoma lesions, particularly in patients with lesions that progress during systemic therapy or are complicated by infection. In this patient, radiotherapy was associated with rapid clinical improvement of the treated lesion. Further studies are needed to better define optimal radiation dosing strategies and treatment sequencing in primary cutaneous anaplastic large cell lymphoma.
